# The Upper Respiratory Tract of Felids Is Highly Susceptible to SARS-CoV-2 Infection

**DOI:** 10.3390/ijms221910636

**Published:** 2021-09-30

**Authors:** Nadine Krüger, Cheila Rocha, Sandra Runft, Johannes Krüger, Iris Färber, Federico Armando, Eva Leitzen, Graham Brogden, Gisa Gerold, Stefan Pöhlmann, Markus Hoffmann, Wolfgang Baumgärtner

**Affiliations:** 1 Infection Biology Unit, German Primate Center, 37077 Göttingen, Germany; crocha@dpz.eu (C.R.); spoehlmann@dpz.eu (S.P.); mhoffmann@dpz.eu (M.H.); 2 Department of Pathology, University of Veterinary Medicine, Foundation, 30559 Hannover, Germany; sandra.runft@tiho-hannover.de (S.R.); Johannes.krueger@tiho-hannover.de (J.K.); iris.verena.faerber@tiho-hannover.de (I.F.); Federico.armando@tiho-hannover.de (F.A.); eva.leitzen@tiho-hannover.de (E.L.); wolfgang.baumgaertner@tiho-hannover.de (W.B.); 3 Department of Biochemistry, University of Veterinary Medicine, Foundation, 30559 Hannover, Germany; graham.brogden@tiho-hannover.de (G.B.); gisa.gerold@tiho-hannover.de (G.G.); 4 Research Center for Emerging Infections and Zoonoses, University of Veterinary Medicine, Foundation, 30559 Hannover, Germany; 5 Institute of Experimental Virology, TWINCORE, Center for Experimental and Clinical Infection Research Hannover, 30625 Hannover, Germany; 6 Wallenberg Centre for Molecular Medicine (WCMM), Umeå University, 90185 Umeå, Sweden; 7 Department of Clinical Microbiology, Umeå University, 90185 Umeå, Sweden; 8 Faculty of Biology and Psychology, Georg-August-University, 37073 Göttingen, Germany

**Keywords:** SARS-CoV-2, felines, respiratory tract, primary cell cultures, ACE2

## Abstract

Natural or experimental infection of domestic cats and virus transmission from humans to captive predatory cats suggest that felids are highly susceptible to SARS-CoV-2 infection. However, it is unclear which cells and compartments of the respiratory tract are infected. To address this question, primary cell cultures derived from the nose, trachea, and lungs of cat and lion were inoculated with SARS-CoV-2. Strong viral replication was observed for nasal mucosa explants and tracheal air–liquid interface cultures, whereas replication in lung slices was less efficient. Infection was mainly restricted to epithelial cells and did not cause major pathological changes. Detection of high ACE2 levels in the nose and trachea but not lung further suggests that susceptibility of feline tissues to SARS-CoV-2 correlates with ACE2 expression. Collectively, this study demonstrates that SARS-CoV-2 can efficiently replicate in the feline upper respiratory tract ex vivo and thus highlights the risk of SARS-CoV-2 spillover from humans to felids.

## 1. Introduction

Severe acute respiratory syndrome coronavirus 2 (SARS-CoV-2) is the causative agent of the coronavirus disease 2019 (COVID-19) and has first been detected in late 2019 in the city of Wuhan, China [1]. Bats harbour viruses closely related to SARS-CoV-2 and are discussed as the natural reservoir host of SARS-CoV-2, whereas pangolins present a potential intermediate host [1,2,3,4,5]. However, other animal species might also contribute to the zoonotic spillover of SARS-CoV-2 from animals to humans. Apart from a variety of felids including cats, lions, tigers, cougars, and snow leopards, different mammalian species such as dogs, ferrets, minks, gorillas, and otters have been identified to be susceptible to natural SARS-CoV-2 infection [6,7,8,9,10,11,12,13,14,15]. With regard to felids, experimental infections have been performed in cats, mainly focusing on viral transmission and clinical signs, showing that SARS-CoV-2 can be transmitted between cats by direct contact or respiratory droplets [16,17,18,19,20]. Thus far, detailed information on virus–host interactions and pathological changes affecting cell morphology are missing for felids. Primary cell cultures present suitable models to study virus–cell interactions in compliance with the 3R (reduction, refinement, replacement) principle. A recent in vitro study showed that SARS-CoV-2 is capable of replicating in well-differentiated cat airway epithelial cells [21]. However, the respiratory tract harbours a variety of different cell types including immune cells, and can, therefore, not be narrowed down to epithelial cells derived from one particular location within the entire respiratory tract.

Here, we focussed on SARS-CoV-2 susceptibility and virus-induced cytological changes of primary cell cultures derived from the upper and lower respiratory tract of domestic cats and a lion. To overcome the restriction to particular cell types or tissues, we employed nasal mucosa explants, tracheal air–liquid interface cultures, and precision-cut lung slices. As angiotensin-converting enzyme 2 (ACE2) constitutes the cellular receptor of SARS-CoV-2, we further investigated ACE2 expression along the respiratory tract of felids and the ability of SARS-CoV-2 spike (S) to mediate entry into cells expressing feline ACE2.

## 2. Results

### 2.1. Replication of SARS-CoV-2 in Primary Cell Cultures Derived from the Upper and Lower Respiratory Tract of Felids

**Figure 1:**
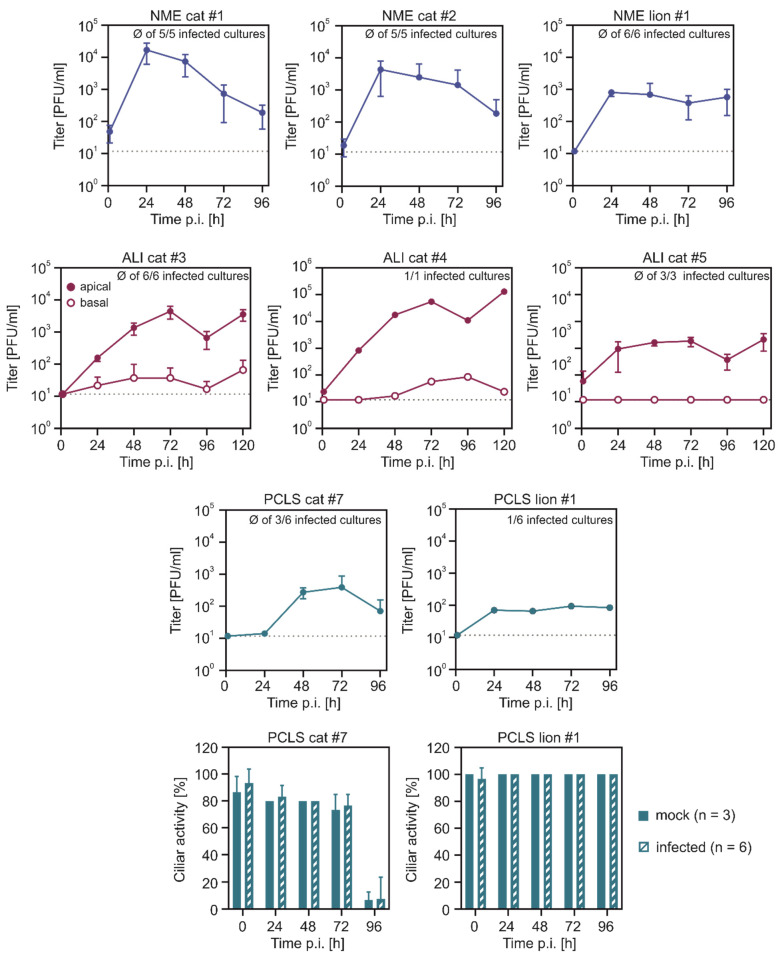
Viral replication of SARS-CoV-2 in feline primary respiratory cell cultures. Nasal mucosa explants (NMEs), air-liquid interface (ALI) cultures derived from the trachea, and precision-cut lung slices (PCLSs) were infected with SARS-CoV-2. Supernatants were collected at the indicated time points and viral titres were determined by titration on Vero E6 cells. Viral titres are given as PFU/mL. Ciliary activity of infected and uninfected PCLSs was semi-quantitatively determined by light microscopy. The graphs show the means and SD of n replicates as indicated in each graph. For viral replication in PCLSs, only slices for which viral replication above the limit of detection has been measured were included in the graph. The dashed lines indicate the limit of detection.

Efficient viral replication of SARS-CoV-2 was observed in NME of cats (cat #1, #2) and lion (lion #1), as indicated by a rapid increase of viral titres within 24 h post infection (p.i.), reaching peak titres between 8 × 10^2^ to 1.6 × 10^4^ PFU/mL (Figure 1). For lion NME, viral titres remained at similar levels as observed for 24 h p.i., whereas for cat NMEs, viral titres decreased during the following days. For cat ALI cultures (cat #3, #4, #5), viral titres increased during 24–72 h p.i. with peak titres of 1.8 × 10^3^ to 5 × 10^4^ PFU/mL, followed by a slight decrease at 96 h p.i. (Figure 1). The decrease of apical titres, together with the slightly increasing basal titres, is most likely a result of impaired epithelial barrier integrity. In contrast, SARS-CoV-2 replication in feline PCLS (cat #7, lion #1) was less efficient. While for NME and ALI cultures, viral replication was observed for all individual NME or transwell filters, only some of the SARS-CoV-2 inoculated PCLS showed viral replication above the limit of detection. Further, peak titres of infected PCLS were approx. 10- to 40-fold lower, compared to ALI cultures or NME. The SARS-CoV-2 infection had no effect on the ciliary activity (Figure 1).

### 2.2. SARS-CoV-2 Infection Does Not Induce Significant Pathomorphological Alterations

For NMEs (cat #1, #2, lion #1) and PCLSs (cat #7, lion #1), H&E examination revealed only minor, non-significant differences between infected and uninfected cultures with regard to intracytoplasmic vacuolisation, nuclear degenerative changes, and hypercellularity in the bronchial epithelium, peribronchial glands, and connective tissue. Infected ALI cultures (cat #5, #6) showed moderate, focal, metaplasia with loss of cilia, as well as moderate amounts of multifocal, clumped, apoptotic cells. Similar changes, except for focal metaplasia, were also seen in the uninfected cultures. Accordingly, statistical assessment of morphological features revealed only minor, non-significant differences between uninfected and infected ALI cultures.

Further, immunolabeling was performed to optimise visualisation of cilia (α-tubulin), macrophages (CD204, not performed in ALI cultures), degradation of cellular proteins (activated caspase-3), and cell proliferation (Ki67) in uninfected and infected NMEs, ALI cultures, and PCLSs. Positively stained cells for all markers were detected in uninfected, as well as infected primary cell cultures, to similar extents, with only minor, non-significant differences between both groups.

**Figure 2:**
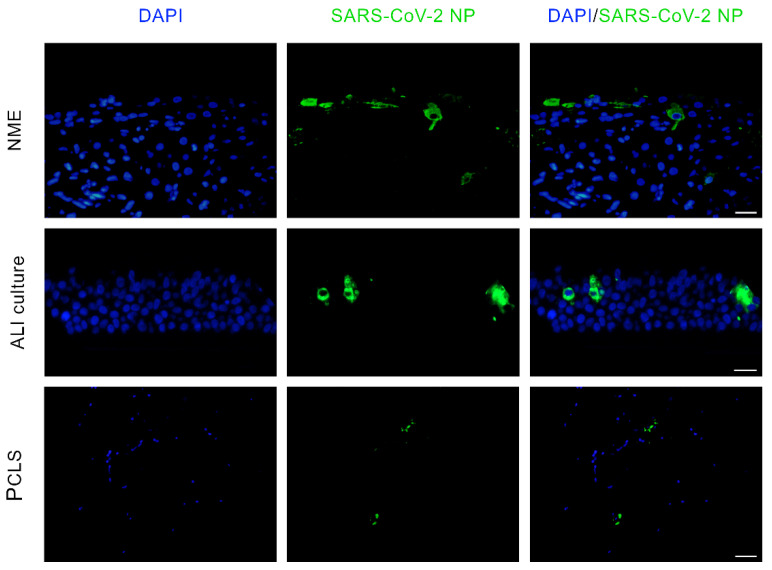
Immunofluorescence staining for SARS-CoV-2 NP in feline primary respiratory cell cultures. SARS-CoV-2 infected and uninfected nasal mucosa explants (NME, cat #2), air–liquid interface (ALI, cat #6) cultures derived from the trachea, and precision-cut lung slices (PCLS, cat #7) were stained for SARS-CoV-2 NP (green) with nuclear counterstaining (blue). Scale bar represents 20 µM.

SARS-CoV-2 NP was detected in NMEs, ALI cultures, and in PCLSs (Figure 2). In ALI cultures, viral antigen was observed multifocally in areas of apoptosis. Similarly, for NMEs and PCLSs, a colocalisation of SARS-CoV-2 NP and apoptotic cells in H&E staining was seen multifocally. Based on the detection of SARS-CoV-2 antigen in respiratory epithelial cells we also investigated whether antigen-positive cells express ACE2. However, the ACE2 specific antibody failed to exhibit immunoreactivity in formalin-fixed, paraffin-embedded tissue. Notably, the same antibody showed positive reactivity in Western blot.

### 2.3. Feline ACE2 Promotes Efficient Entry of SARS-CoV-2

**Figure 3:**
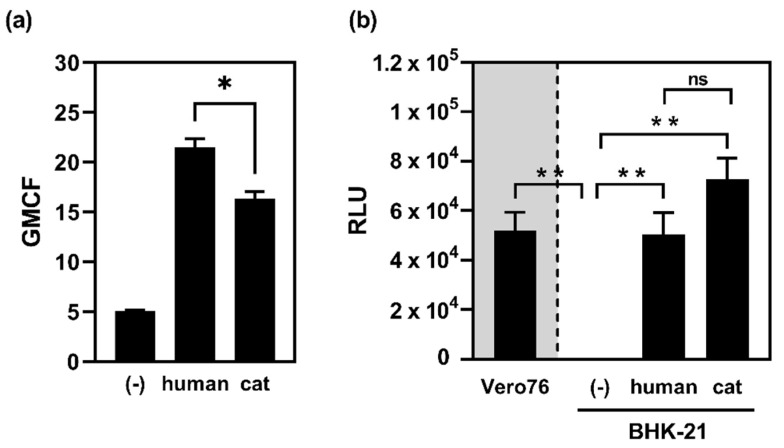
Feline ACE2 facilitates SARS-CoV-2 S-mediated entry: (**a**) surface expression of transiently expressed ACE2. BHK-21 cells were transfected for the expression of human or cat ACE2, empty vector served as control. ACE2 was stained by polyclonal antibodies directed against ACE2 and anti-goat Alexa Fluor^®^488-conjugated secondary antibodies. ACE2 expression was quantified by flow cytometry and is given as geometric mean channel fluorescence (GMCF). The graph shows the means and SEM of three independent experiments. Statistical significance was determined by paired, two-tailed *t*-test. *: *p* ≤ 0.05; (**b**) ACE2 expressing Vero E6 cells and BHK-21 cells lacking ACE2 expression served as positive and negative controls, respectively. Prior to transduction, BHK-21 cells were transfected for the expression of human or cat ACE2. Transduction was quantified by measuring luciferase activity and is given as relative luminescence units (RLU). The graph shows the means and SEM of three independent experiments performed as quadruplicates. Statistical significance was determined by paired, two-tailed *t*-test. **: *p* ≤ 0.01.

To analyse whether feline ACE2 facilitates S-driven entry as efficient as human ACE2, rhabdoviral pseudotypes were equipped with SARS-CoV-2 S. Pseudotype entry was observed for Vero E6 but not for BHK-21 cells, a cell line known to be negative for the expression of ACE2 and, therefore, not supporting SARS-CoV-2 S-driven entry [22,23,24]. The exogenous expression of human or cat ACE2 led to robust ACE2 surface expression, with human ACE2 being slightly better (~1.3-fold) expressed (Figure 3a), and rendered BHK-21 cells susceptible to S-mediated entry (Figure 3b).

**Figure 4:**
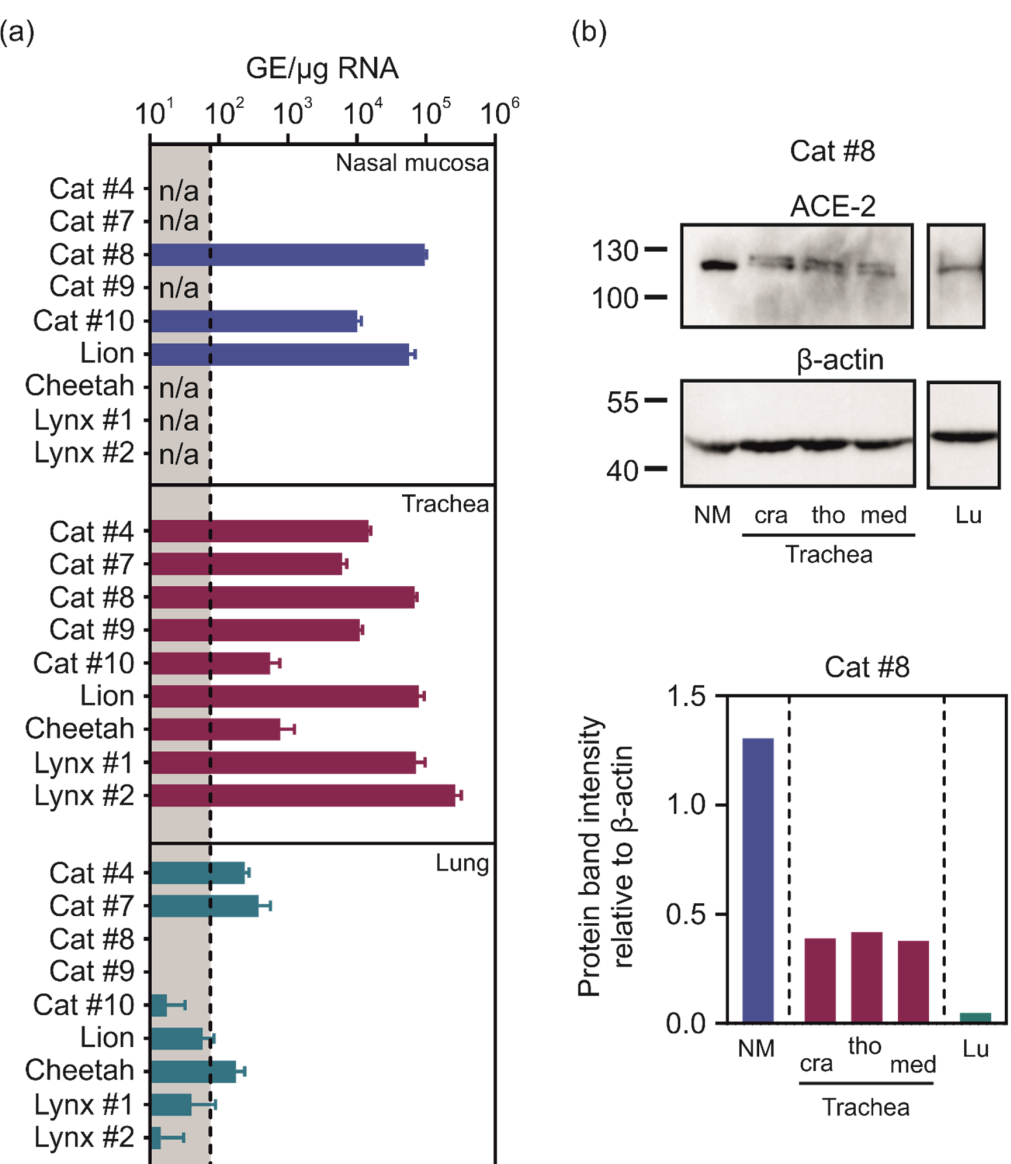
Expression of ACE2 in the respiratory tract of felines. (**a**) mRNA expression of ACE2 in lung, trachea, and nasal mucosa. RNA was extracted from organ samples, followed by cDNA synthesis and qPCR targeting cat ACE2. Cycle threshold (ct) values were normalised to total RNA. Expression plasmids containing feline ACE2 were used to generate a standard curve to calculate the amounts of genomic equivalents (GE) based on the ct values. The graph shows the means and SD of trials measured as quintuplicates. n/a: no organ sample available. The dashed line indicates the background; (**b**) detection of ACE2 and β-actin in nasal mucosa (NM), cranial (cra), median (med), and thoracic (tho) parts of the trachea, and lung (Lu). Numbers indicate molecular weight [kDa]. For quantification, protein band intensity for ACE2 was set in correlation to the corresponding β-actin bands.

In order to analyse the distribution of ACE2 within the feline respiratory tract, organ samples derived from nasal mucosa, trachea, and lungs of cat, lion, cheetah, and lynx were analysed for mRNA expression levels of ACE2. Despite individual variations, a similar pattern was obtained for all felids: High amounts of genomic ACE2 copies (1.0 to 9.6 × 10^4^ GE of ACE2/µg RNA) were detected in tissues derived from nasal mucosa (Figure 4a). The expression levels of ACE2 mRNA within the nasal tissue were at least comparable or even higher than those detected in trachea samples of the same animals. In contrast, ACE2 mRNA was barely detected in lung tissue. In order to analyse whether the differences obtained for the mRNA expression of ACE2 reflect the levels of protein expression, Western blotting was performed. In accordance with the results obtained by qPCR, high amounts of ACE2 protein were detected in the nasal mucosa, followed by the trachea, whereas less ACE2was detected in lung tissue lysates (Figure 4b). Interestingly, an ACE2 double band was detected in trachea samples, indicative of differential posttranslational modification of ACE2 in the feline respiratory tract. Within the trachea, no differences in protein expression of ACE2 were observed for the cervical, median, and thoracic parts.

## 3. Discussion

Felids have been discussed as suitable intermediate hosts of SARS-CoV-2, as they are highly susceptible under natural conditions [7,8,9,15,25]. Thus far, there is no evidence of SARS-CoV-2 circulation in domestic cats or zoonotic virus transmission from felids to humans [26]. However, mink, another susceptible animal species, have been shown to transmit the virus to humans [27]; therefore, future spillover events from animals to humans cannot be excluded. Experimental infections showed that cats most likely do not develop a clinical illness or only mild symptoms, although viral replication and shedding via the respiratory and gastrointestinal tract has been observed [16,17,18,19,20,28]. Accordingly, only mild-to-moderate pathological changes such as interstitial pneumonia and bronchiolitis were detected in the respiratory tract of experimentally infected cats [19,28], whereas severe pathological alterations including necrotic changes were observed in human patients with lower respiratory tract infections [29,30]. It has been suggested that deficiencies of key components of the inflammasome and pyroptosis pathway are responsible for the mild symptomatology observed in cats [31].

Two studies showed that viral titres and/or RNA levels in the lungs of experimentally infected cats tended to be lower compared to samples derived from the upper respiratory tract [16,19]. According to these observations, viral replication was highly efficient in nasal mucosa explants, as indicated by a rapid increase of viral titres within 24 h, whereas lung slices were barely susceptible to SARS-CoV-2 infection. In agreement with a previous study [21], well-differentiated feline tracheal ALI cultures were susceptibly to SARS-CoV-2 infection although the replication was delayed, compared to NMEs.

ACE2 plays a crucial role in SARS-CoV-2 entry as it serves as the main cellular receptor for viral attachment [22]. To explain the differences observed regarding the susceptibility of the primary cell cultures and explants, we investigated the expression of ACE2 and its usage as a cellular receptor for SARS-CoV-2. Among feline species, the amino acid sequence of ACE2 is highly conserved, and human and feline ACE2 share a homology of 85% in total and 80% referring to the residues involved in SARS-CoV-2 attachment (Supplementary Material Figure S1a). In consistence with previous studies [32,33,34], we showed that feline ACE2 is capable of mediating SARS-CoV-2 S-driven entry as efficiently as human ACE2. Investigations on the expression levels of ACE2 within the respiratory tract of felids revealed that the highest amounts of ACE2 mRNA and protein were detected in nasal mucosa, whereas only limited expression was observed in lung tissue. Therefore, we assume that the availability of ACE2 plays an important role in the susceptibility of respiratory tract tissues of felids and might explain the differences in viral replication within cells derived from the nose, trachea, and lung. Thus far, it is unknown whether the expression of ACE2 in felids is up-or downregulated by manifold factors as described for humans such as cigarette smoke [35,36] or chronic diseases and allergies of the respiratory tract [37,38,39]. In addition to binding to the cellular receptor ACE2, SARS-CoV-2 entry involves the proteolysis of the spike protein by the transmembrane protease TMPRSS2 [22]. For humans, it has been assumed that most likely the availability of ACE2, rather than TMPRSS2, might limit SARS-CoV-2 entry due to the broader and more robust expression of TMPRSS2 [40]. Further, it has been shown that ACE2 is more variable among mammalian species, compared to the highly conserved protease TMPRSS2, suggesting that ACE2 compatibility plays the main role during inter-species transmission [41]. For TMPRSS2, amino acid residues forming the catalytically centre (H296, D345, S441) and substrate binding site (D435, S460, G462), as well as amino acid residues involved in intermolecular interactions between TMPRSS2 and the SARS-CoV-2 S cleavage site, have been identified [42]. Since these residues are highly conserved between human and feline TMPRSS2 (Supplementary Material Figure S1b), it is very likely that feline TMPRSS2 is capable of efficiently activating SARS-CoV-2 S. Similar to the observations derived for human tissues [40], Chen et al. reported that the expression levels of TMPRSS2 in cat lungs exceeded those of ACE2 especially in ciliated and secretory cells [43]. In cats, co-expression of ACE2 and TMPRSS2 was observed in cells from the lung, kidney, eyelid, oesophagus and rectum; moreover, the number of ACE2/TMPRSS2 co-expressing cells in feline tissues was higher as compared to other vertebrate species [43].

Based on our findings, we assume that SARS-CoV-2 most likely infects the upper respiratory tract in felines and might, therefore, facilitate an easy spread of infection via nasal discharge. Further, we show that the combination of primary cell cultures derived from different regions of the respiratory tract is a suitable tool to investigate SARS-CoV-2 susceptibility and host–cell interactions, as our results are in compliance with previously published in vivo studies. The usage of primary cell culture models complies with the 3R principles and also enables studies focusing on SARS-CoV-2 susceptibility of endangered or exotic species that cannot be used for in vivo studies due to legal bases or factors impeding their husbandry and care. After having shown that feline primary cell culture systems are susceptible to SARS-CoV-2 infections in general, future studies will address the questions of whether emerging SARS-CoV-2 variants differ in viral replication and induction of pathomorphological changes in the feline respiratory tract cultures. Further, as the infection of new host species usually causes a virus to mutate in order to adapt to efficient usage of host cell factors, the emergence of adaptive mutations in the SARS-CoV-2 genome, especially in the spike protein, following replication in feline cells warrants further studies.

A limitation of our study is that primary cell culture models and tissue explants cannot reflect all aspects of SARS-CoV-2 infection and pathogenesis with respect to the route of infection, virus dissemination within the organism, and host immune response. Our study intended to investigate the susceptibility of different tissues within the respiratory tract to SARS-CoV-2 infections. By using immortalised cell lines, it has been shown that SARS-CoV-2 entry depends on the presence of suitable host cell receptors and the proteolytic processing of the spike protein by host cell proteases [22,44]. As immortalised cells do not necessarily reflect the in vivo situation regarding expression pattern and levels of essential host cell factors, authentic tissue and organ samples are indispensable to evaluate the susceptibility of particular tissues and hosts. Given that our findings regarding viral replication within the upper and lower respiratory tract of cats were in accordance with data obtained by in vivo infection studies [16,19], primary cell cultures represent a suitable model to study viral entry and virus–host interactions of SARS-CoV-2.

## 4. Materials and Methods

### 4.1. Cell Culture

Vero E6 (ATCC: CRL-1586) and BHK-21 (ATCC: CCL-10) cells were maintained in Dulbecco’s modified Eagle´s medium (DMEM; PAN-Biotech, Aidenbach, Germany) supplemented with 5% fetal calf serum (FCS; Biochrom, Berlin, Germany) and incubated at 37 °C and 5% CO_2_.

### 4.2. Collection of Felid Samples

Tissue samples were collected from feline predators and domestic cats of various ages, sexes, and breeds, brought to the Department of Pathology, University of Veterinary Medicine, Hannover, for postmortem necropsy (Table 1). Samples used for the generation of primary cell cultures were collected from necropsy cases that were free of respiratory diseases. Organ samples for qPCR and Western blotting included kidney, nasal mucosa, trachea, and lung.

### 4.3. Generation of Primary Cell Cultures

Primary cell cultures investigated in this study include nasal mucosa explants (NME), air–liquid interface (ALI) cultures, and precision-cut lung slices (PCLS). All primary cell cultures were maintained at 37 °C and 5% CO_2_.

To generate NME from the nasal septum, the respiratory mucosa was stripped from the underlying cartilage and washed with PBS and a mild disinfectant solution (Prontosan^®^C; B. Braun, Melsungen, Germany). Next, the respiratory mucosa was cut into pieces about 16–25 mm^2^ in size and placed on semipermeable transwell membranes (pore size: 0.4 µm; VWR, Radnor, PA, USA) with the epithelial surface facing upwards. Explants were maintained at an air–liquid interface with DMEM supplemented with penicillin–streptomycin (10,000 U/mL penicillin, 10 mg/mL streptomycin; Sigma-Aldrich, St. Louis, MO, USA), enrofloxacin (50 mg/mL; Bayer, Leverkusen, Germany), and amphotericin B (250 µg/mL; Sigma-Aldrich, St. Louis, MO, USA).

ALI cultures were generated as described previously [45]. Briefly, the trachea was washed with PBS, followed by enzymatic digestion with protease and desoxyribonuclease I (Sigma-Aldrich, St. Louis, MO, USA) for 24 h at 4 °C. Epithelial cells were scraped from the tissue, washed with PBS, collected by centrifugation at 250× *g* for 10 min, and purified using a cell strainer, before the cells were seeded onto type I collagen (Sigma-Aldrich, St. Louis, MO, USA) coated flasks. After reaching 70–80% confluence, cells were harvested from the flasks, centrifuged at 250× *g* for 10 min and resuspended in ALI medium, consisting of DMEM and Bronchial Epithelial Cell Growth Basal Medium (BEBM; Clonetics, San Diego, CA, USA) mixed at a ratio of 1:1 and supplements (Supplementary Material Table S1). Cells were seeded on the apical side of type IV collagen (Sigma-Aldrich, St. Louis, MO, USA) coated, semipermeable transwell membranes (pore size: 0.4 µm; VWR, Radnor, PA, USA) at a density of 0.35 million cells per membrane and 500 µL ALI medium was added to the basolateral compartment. The medium was changed every 48 h and transepithelial electrical resistance (TEER) was measured daily to verify the integrity of cellular barriers. After seven days, ALI conditions were initiated by adding medium only to the basolateral compartment. Medium changes were continued every 48 h and once a week, TEER was measured. After approx. 28 days of culturing under ALI conditions, primary cell cultures were subjected to infection studies. No suitable tissue material was available for the generation of lion ALI cultures.

PCLS were prepared as described previously [46,47]. Briefly, 1.5% low-melting agarose (Gerbu, Heidelberg, Germany) was dissolved in RPMI (Thermo Fisher Scientific, Waltham, MA, USA)/Aqua bidest (1:1 mixture) and heated to 37 °C. Next, the main bronchus of each lobe was filled with liquid agarose and put on ice. After solidification, lung lobes were cut into small cylinders with 8 mm in diameter using a coring press (Alabama R&D, Munford, AL, USA), then trimmed to equally thick slices of ~250 µm with a Krumdieck tissue slicer (Alabama R&D, Munford, AL, USA). Slice thickness was measured using a tissue slice thickness gauge (Alabama R&D, Munford, AL, USA). PCLS were washed three times with DMEM/F12 without phenol red (Thermo Fisher Scientific, Waltham, MA, USA) substituted with penicillin–streptomycin, enrofloxacin, and amphotericin B to remove remaining agarose before they were transferred to 24-transwell plates with 500 µL DMEM/F12 per well. The vitality of PCLS was monitored by observing the ciliary activity of the ciliated cells of the bronchi via light microscopy.

### 4.4. SARS-CoV-2 Infection of Primary Respiratory Cell Cultures

All work with infectious SARS-CoV-2 was conducted under BSL-3 conditions at the German Primate Centre, Göttingen/Germany. NME and ALI cultures were infected from the apical side with 1 × 10^4^ plaque-forming units (PFUs) of SARS-CoV-2 isolate NK, Pango lineage B.1.513 (kindly provided by Stephan Ludwig, Institute of Virology, University of Münster, Münster, Germany), in an inoculation volume of 100 µL. PCLSs were infected with 1 × 10^5^ PFU in an inoculation volume of 250 µL. After 1 h, the inoculum was removed, cell cultures were washed with PBS three times, and 500 µL of culture medium was added to the basal side of NME and ALI cultures and each PCLS. For harvesting supernatants from PCLSs and the basal side of ALI cultures, 100 µL of culture medium was collected and subsequently replaced by the same volume of fresh medium. For collecting newly released viral particles from the apical side of ALI cultures and NMEs, 100 µL medium were added on top of the transwell filters followed by incubation at 37 °C for 10 min. Next, the apical side was rinsed with the medium three times before the virus-containing medium was stored at -80 °C until further usage. The ciliary activity of PCLS was evaluated on a daily basis by light microscopy using a Keyence BZ-X800 microscope (Keyence, Osaka, Japan). Viral titres were determined by plaque assay on VeroE6 cells and are given as PFU/mL as described previously [48].

### 4.5. Histologic Evaluation of Primary Cell Cultures

Infected primary cultures and uninfected controls were washed three times with PBS and fixed in 10% formalin. For histological examination, formalin-fixed samples were embedded in paraffin wax, and 2 μm thick serial sections were processed and stained with haematoxylin and eosin (H&E). ALI cultures and their respective membrane were sliced in two halves in order to be able to evaluate a broader section of the cultured respiratory epithelium.

The number of ciliated cells and cells with intracytoplasmic vacuoles and nuclear degenerative changes in the epithelium of the NMEs were counted and compared to the total number of epithelial cells. For the submucosal glands and connective tissue, cells in hypercellular areas, cells with intracytoplasmic vacuoles, or nuclear degenerative changes were counted and compared to the number of evaluated high power fields (hpf, 400× magnification).

To evaluate ALI cultures, the two halves of each sample were evaluated by counting the number of ciliated cells, intracytoplasmic vacuoles, and nuclear degenerative changes in each hpf. Afterwards, absolute values were converted into percent values in relation to the total number of cells within each hpf.

For PCLS, cells in hypercellular areas and cells with intracytoplasmic vacuoles and nuclear degenerative changes were counted separately in different compartments (epithelium, peribronchial glands, connective tissue of the bronchi and bronchioles, alveolar epithelium, interstitium). Furthermore, ciliated bronchial epithelial cells and cells in the alveolar lumen were counted. For the bronchial epithelium, cells with morphological changes were set in relation to the total number of bronchial epithelial cells. For the other compartments, cells showing changes were divided by the number of evaluated hpf.

In addition, immunohistochemistry was performed on formalin-fixed, paraffin-embedded (FFPE) samples using the avidin–biotin–peroxidase complex (ABC; Vector Laboratories, Burlingame, CA, USA) method as previously described [49,50]. Primary antibodies targeting caspase-3, CD204 (PCLS), α-tubulin, and ki67 and secondary antibodies are listed in Supplementary Material Table S2. Positive controls for primary antibodies used in immunohistochemistry were performed contemporaneously on specimens containing the target molecule from the analysed species listed in Supplementary Material Table S2. Incubation with antibodies was carried out overnight at 4 °C. Positively stained cells were divided by the number of evaluated hpf for NMEs. In ALI cultures, the number of positively stained cells for each hpf was counted and set in relation to the total number of cells, which was counted in the H&E sample. For the PCLS, five representative hpf, including alveolar areas and bronchi, were counted.

### 4.6. Immunofluorescence Microscopy

NME, ALI, and PCLS FFPE samples were stained as previously described [51]. Briefly, FFPE tissue sections were deparaffinised, rehydrated through graded alcohol, and pretreated for antigen retrieval using citrate buffer. Following the blocking of unspecific bindings with goat serum, sections were incubated with primary antibodies targeting SARS-CoV-2 nucleoprotein (NP) and secondary antibodies (Supplementary Material Table S2) for 90 and 60 min at room temperature, respectively. Slides were stained for cell nuclei and mounted using Fluoroshield^TM^ mounting medium (Sigma-Aldrich, St. Louis, MO, USA) and coverslips. Sections were screened for the presence of SARS-CoV-2 NP using a fluorescence microscope (Olympus IX-70, Olympus Optical Co. GmbH, Tokyo, Japan) equipped with an Olympus DP72 camera and Olympus cell sense standard software version 2.3.

### 4.7. RNA Isolation and qPCR

A total of 50–100 mg of organ samples from nasal mucosa, trachea, or lung was mechanically disrupted by cutting before 1 mL TRIzol reagent (Invitrogen, Waltham, MA, USA) was added. Samples were incubated for 5 min at room temperature, followed by homogenisation using a bead-beating tissue homogeniser. RNA was extracted using TRIzol reagent. RNA was used as a template for cDNA synthesis using the SuperScript III First-Strand Synthesis System and random hexamers (Thermo Fisher Scientific, Waltham, MA, USA). Finally, cDNA was subjected to qPCR using SYBR Green Mastermix (Thermo Fisher Scientific, Waltham, MA, USA), and primers targeting cat ACE2 (for: CAAGCACTTACAATTGTTGGAA, rev: TGAGTAATCATTAGCAACATGGAA). Cycle threshold (ct) values were normalised to total RNA. Dilution series of expression plasmids containing cat ACE2 were used as standards to calculate the amounts of genomic equivalents (GE) based on the ct values.

### 4.8. Detection of ACE2 by Western Blotting

Tissue samples were mechanically disrupted by homogenisation using a bead-beating tissue homogeniser after adding lysis buffer (50 mM Hepes, pH 7.4, 150 mM NaCl, 10% glycerol, 1% NP-40, 1 mM CaCl_2_) containing protease and phosphatase inhibitors (Sigma-Aldrich, St. Louis, MO, USA). Immunoblotting was carried out as formerly described [52]. Briefly, 100 µg of each sample was separated on an 8% SDS–PAGE gel. The reaction was performed using antibodies as indicated in Supplementary Material Table S2. In advance, tissue homogenate derived from the kidney was used as a positive control. Protein bands were visualised using SuperSignal™ West Femto maximum sensitivity Western blot chemiluminescence substrate (Thermo Fisher Scientific, Waltham, MA, USA) and a ChemiDoc MP Imaging System (Bio-Rad, Hercules, CA, USA). ACE2 band intensities were quantified via ImageJ v8 and normalised against β-actin.

### 4.9. Transduction Experiments

Vesicular stomatitis virus (VSV) pseudotypes harbouring the SARS-CoV-2 spike protein (S) were generated as described previously [22]. BHK-21 cells were transfected for the expression of human [53] or cat ACE2 that has been cloned from cDNA generated from trachea samples as described above, using Lipofectamine 2000 transfection reagent (Thermo Fisher Scientific, Waltham, MA, USA). At 24 h post transfection (p.t.), cells were inoculated with SARS-CoV-2 S-pseudotyped VSV for 16–18 h. ACE2 expressing Vero E6 cells and BHK-21 cells transfected with empty plasmid were used as positive and negative controls, respectively. To quantify the efficiency of S-mediated pseudotype entry, cells were lysed with 1× Cell Culture Lysis Reagent (Promega, Madison, WI, USA) for 30 min at room temperature. Lysates were transferred into white 96-well plates and firefly luciferase activity was measured using Beetle-Juice substrate (PJK, Kleinblittersdorf, Germany) and a Hidex Sense plate luminometer (Hidex, Turku, Finland).

### 4.10. Flow Cytometry Analysis

BHK-21 cells were transfected for the expression of empty vector pCG1, human or cat ACE-2_using Lipofectamine 2000 transfection reagent (Thermo Fisher Scientific, Waltham, MA, USA). At 48 h p.t., cells were washed with PBS, scraped from the bottom of the wells, and transferred into reaction tubes. Cells were collected by centrifugation, followed by a second washing step with PBS. After centrifugation, the cell pellet was resuspended in BSA containing polyclonal anti-human ACE-2 antibodies (dilution: 1:200; R&D Systems, #AF933) and incubated on an overhead shaker for 1 h at 4 °C. Next, cells were centrifuged and washed with PBS before they were incubated with anti-goat IgG Alexa Fluor 488 (dilution: 1:500; Invitrogen) again for 1 h. After incubation, cells were washed with PBS, resuspended in BSA, and subjected to flow cytometry analysis using a BD LSR II flow cytometer (BD Biosciences, Franklin Lakes, NJ, USA).

### 4.11. Statistical Analysis

Statistical analyses were performed using GraphPad Prism software version 9.1.0 and SPSS software (IBM SPSS Statistics 26; IBM, Armonk, NY, USA). Statistical significance of transient ACE2 expression and pseudoviral transduction efficiency was determined by paired, two-tailed *t*-test and confidence intervals of 95%. Additionally, data obtained from the histological evaluation were analysed using Shapiro–Wilk normality tests, followed by Mann–Whitney U tests. Differences between groups as detected by Mann–Whitney U tests were considered significant at a *p*-value of <0.05.

## Figures and Tables

**Table 1:** Collection of felid samples for the generation of primary cultures, pathological, and biochemical examinations.

Species	Case No.	Primary Cell Culture	Investigations	n_inf_/n_rep_
Cat (*Felis catus*)	#1	NME	Virus replication, IF, IHC	5/5
	#2	NME	Virus replication, IF, IHC	5/5
	#3	ALI	Virus replication, IF	6/6
	#4	ALI	Virus replication, qPCR	1/1
	#5	ALI	Virus replication, IF, IHC	3/3
	#6	ALI	IF, IHC	-
	#7	PCLS	Virus replication, qPCR, IF, IHC	6/3
	#8	-	qPCR, WB	-
	#9	-	qPCR	-
	#10	-	qPCR	-
Lion (*Panthera leo leo*)	#1	NME, PCLS	Virus replication, qPCR, IF, IHC	6/6 (NME), 6/1 (PCLS)
Cheetah (*Acinonyx jubatus*)	#1	-	qPCR	-
Lynx (*Lynx rufus*)	#1	-	qPCR	-
	#2	-	qPCR	-

NME: nasal mucosa explant, ALI: air–liquid interface cultures, PCLS: precision-cut lung slices, IHC: immunohistochemistry, IF: immunofluorescence microscopy, WB: western Blotting, ninf: number of NME/ALI/PCLS inoculated with SARS-CoV-2, nrep: number of inoculated cultures with replication above the limit of detection.

## Data Availability

The data presented in this study are available on request from the corresponding author.

## Supplementary Materials

The following are available online at https://www.mdpi.com/article/10.3390/ijms221910636/s1.
