# Supplementary material for: The Upper Respiratory Tract of Felids Is Highly Susceptible to SARS-CoV-2 Infection
Source: Int J Mol Sci. 2021 Sep 30;22(19):10636. doi: 10.3390/ijms221910636 (PMC8508926; doi:10.3390/ijms221910636)
Supplement: Supplementary file 1 [file ijms-22-10636-s001.zip › ijms-1388757-supplementary.pdf]

Fig. S1

a)

ACE2	24	27	28	30	31	34	35	37	38	41	42	79	82	83	330	353	354	355	357	393
Homo sapiens	**Q**	**T**	**F**	**D**	**K**	**H**	E	**E**	**D**	**Y**	**Q**	**L**	**M**	**Y**	**N**	**K**	**G**	**D**	**R**	R
Acinonyx jubatus	L	.	.	E	.	.	.	.	E	.	.	.	T	.	.	.	.	.	.	.
Felis catus	L	.	.	E	.	.	.	.	E	.	.	.	T	.	.	.	.	.	.	.
Lynx canadensis	L	.	.	E	.	.	.	.	E	.	.	.	T	.	.	.	.	.	.	.
Neofelis diardi	L	.	.	E	.	.	.	.	E	.	.	.	T	.	.	.	.	.	.	.
Neofelis nebulosa	L	.	.	E	.	.	.	.	E	.	.	.	T	.	.	.	.	.	.	.
Panthera pardus	L	.	.	E	.	.	.	.	E	.	.	.	T	.	.	.	.	.	.	.
Panthera tigris	L	.	.	E	.	.	.	.	E	.	.	.	T	.	.	.	.	.	.	.
Puma yagouaroundi	L	.	.	E	.	.	.	.	E	.	.	.	T	.	.	.	.	.	.	.
Puma concolor	L	.	.	E	.	.	.	.	E	.	.	.	T	.	.	.	.	.	.	.

b)

TMPRSS2	275	276	278	280	296	299	300	338	340	341	342	345	387	388	389	390
Homo sapiens	**V**	**Q**	**V**	**V**	**H**	**E**	K	**D**	**K**	**T**	**K**	**D**	**T**	**E**	**E**	**K**
Acinonyx jubatus	.	.	.	.	.	.	E	.	.	.	.	.	.	H	.	.
Felis catus	.	.	.	.	.	.	E	.	.	.	.	.	.	H	.	.
Lynx canadensis	.	.	.	.	.	.	E	.	.	.	.	.	.	H	.	.
Panthera leo	.	.	.	.	.	.	E	.	.	S	.	.	.	H	.	.
Panthera pardus	.	.	.	.	.	.	E	.	.	S	.	.	.	H	.	.
Panthera tigris	.	.	.	.	.	.	E	.	.	S	.	.	.	H	.	.
Puma yaqouaroundi	.	.	.	.	.	.	E	.	.	.	.	.	.	H	.	.

TMPRSS2	391	392	413	414	419	431	433	435	438	441	460	462	465	466	467	469
Homo sapiens	**G**	**K**	**R**	**Y**	**L**	**Q**	N	**D**	**Q**	**S**	**S**	**G**	**C**	**A**	**K**	**Y**
Acinonyx jubatus	.	.	K	.	.	.	T	.	.	.	.	.	.	.	.	N
Felis catus	.	.	K	.	.	.	T	.	.	.	.	.	.	.	.	N
Lynx canadensis	.	.	K	.	.	.	T	.	.	.	.	.	.	.	.	N
Panthera leo	.	.	K	.	.	.	T	.	.	.	.	.	.	.	.	N
Panthera pardus	.	.	K	.	.	.	T	.	.	.	.	.	.	.	.	N
Panthera tigris	.	.	K	.	.	.	T	.	.	.	.	.	.	.	.	N
Puma yaqouaroundi	.	.	K	.	.	.	T	.	.	.	.	.	.	.	.	N

Comparision of amino acid residues of human and feline ACE2 (a) and TMPRSS2 (b) involved in SARS-CoV-2 interactions. GenBank accession numbers: *H. sapiens*, ACE2: BAB40370.1, TMPRSS2: AAD37117.1; *A. jubatus*, ACE2: XP_026910297.1, TMPRSS2: XP_026897377.1; *F. catus*, ACE2: AAX59005.1, TMPRSS2: XP_023094479.1; *L. canadensis*, ACE2: XP_030160839.1, TMPRSS2: XP_030185865.1, *N. diardi*, ACE2: QNC68916.1; *N. nebulosa*, ACE2: QNC68918.1; *P. leo*, TMPRSS2: XP_042812026.1; *P. pardus*, ACE2: XP_019273509.1, TMPRSS2: XP_019275943.1; *P. tigris*, ACE2: XP_007090142.2, TMPRSS2: XP_042812429.1; *P. yagouaroundi*, ACE2: XP_040324139.1, TMPRSS2: XP_040339813.1; *P. concolor*, ACE2: XP_025790417.1. Red letters indicate catalytically active site residues of TMPRSS2.

Table S1. Cell culture medium supplements for maintenance of ALI cultures.

Additives	Company	Initial concentration	Final concentration
Bovine serum albumin	Sigma-Aldrich	150 mg/ml	500 µg/ml
Bovine pituitary extract	Sigma-Aldrich	14 mg/ml	14 µg/ml
Insulin	Sigma-Aldrich	10 mg/ml	5 µg/ml
Transferrin	Sigma-Aldrich	10 mg/ml	20 µg/ml
Hydrocortisone	Sigma-Aldrich	0.072 mg/ml	72 ng/ml
Triiodothyronine	Sigma-Aldrich	0.067 mg/ml	67 ng/ml
Epinephrine	Sigma-Aldrich	0.6 mg/ml	0.6 µg/ml
Epidermal Growth Factor	BD Biosciences	50 µg/ml	50 ng/ml (BEGM)<br><br>1 ng/ml ALI
Retinoic Acid	Sigma-Aldrich	5 x 10^{-5} mM/ml	0.001 nM/ml
Phosphorylethanolamine	Sigma-Aldrich	70 mg/ml	70 µg/ml
Ethanolamine	Sigma-Aldrich	30 µg/ml	30 ng/ml
Penicillin/Streptomycin	Sigma-Aldrich	10000 U/ml penicillin,<br>10 mg/ml streptomycin	1%
Fetal calf serum	Capricorn	10%	10%
Enrofloxacin	Bayer Animal Health	50 mg/ml	50 mg/ml

Table S2. Antibodies used for immunohistochemistry (IHC), immunofluorescence microscopy (IF) and western Blotting (WB).

Target, clonality, host	Application	Dilution	Positive controls	Supplier, Catalog No.
Caspase 3, polyclonal, rabbit	IHC	1:200	Lymph node	Promega, G7481
CD204, monoclonal, mouse	IHC	1:500	Lymph node	Abnova Corporation, MAB1710
α-tubulin, monoclonal, mouse	IHC	1:100	Trachea	Sigma-Aldrich, T6793
Ki67, clone MIB-1, monoclonal, mouse	IHC	1:100	Intestine	Dako Cytomation, GA62661-2
SARS-CoV-2 NP, clone #5, monoclonal, mouse	IF	1:500	Lung from SARS-CoV-2 infected hamsters	SinoBiological, 40143-MM05
ACE2, clone SN0754, monoclonal, rabbit	WB	1:5000	Kidney	NovusBio, NBP2-67692
β-actin HRP, clone AC-15, monoclonal, mouse	WB	1:10000	-	Sigma-Aldrich, A3854
Goat anti-rabbit IgG-b	IHC	1:200	-	Vector Laboratories, BA-1000
Goat anti-mouse IgG	IHC	1:200	-	Vector Laboratories, BA-9200
ALEXA Fluor® 488 goat anti-mouse IgG	IF	1:200	-	Invitrogen, A28175
Goat anti-rabbit IgG HRP	WB	1.5000	-	Invitrogen, 31460
